# Cohesion Gain Induced by Nanosilica Consolidants for Monumental Stone Restoration

**DOI:** 10.1021/acs.langmuir.2c00486

**Published:** 2022-05-23

**Authors:** Joanna Dziadkowiec, Hsiu-Wei Cheng, Michael Ludwig, Matea Ban, Timon Pascal Tausendpfund, Regine von Klitzing, Markus Mezger, Markus Valtiner

**Affiliations:** † NJORD Centre, Department of Physics, University of Oslo, Oslo 0371, Norway; ‡ Institute of Applied Physics, Applied Interface Physics, Vienna University of Technology, Vienna 1040, Austria; § Soft Matter at Interfaces, Department of Physics, Technical University of Darmstadt, 64289 Darmstadt, Germany; ∥ Materials Testing Institute, University of Stuttgart, 70569 Stuttgart, Germany; ⊥ Max Planck Institute for Polymer Research, 55128 Mainz, Germany; # Dynamics of Condensed Systems, Department of Physics, University of Vienna, 1090 Wien, Austria

## Abstract

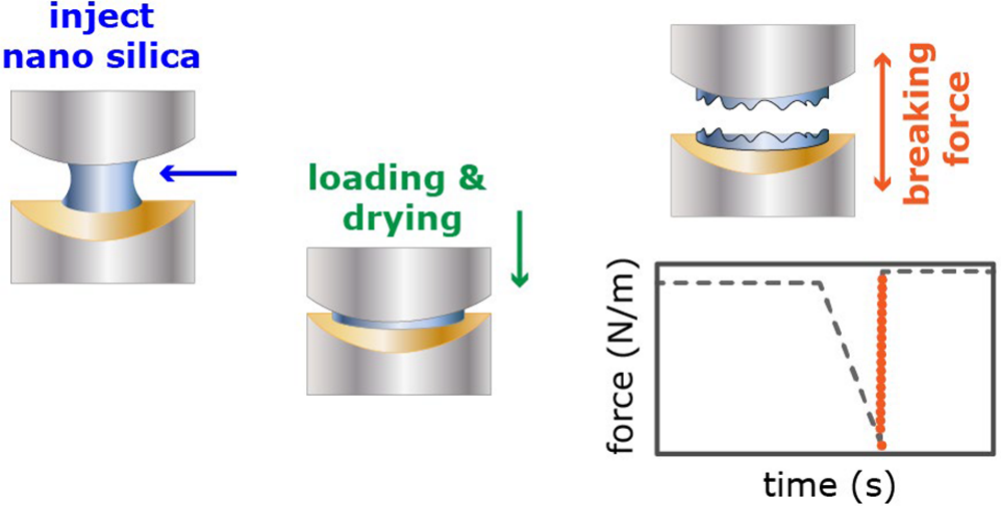

Mineral nanoparticle suspensions with consolidating properties have been successfully applied in the restoration of weathered architectural surfaces. However, the design of these consolidants is usually stone-specific and based on trial and error, which prevents their robust operation for a wide range of highly heterogeneous monumental stone materials. In this work, we develop a facile and versatile method to systematically study the consolidating mechanisms in action using a surface forces apparatus (SFA) with real-time force sensing and an X-ray surface forces apparatus (X-SFA). We directly assess the mechanical tensile strength of nanosilica-treated single mineral contacts and show a sharp increase in their cohesion. The smallest used nanoparticles provide an order of magnitude stronger contacts. We further resolve the microstructures and forces acting during evaporation-driven, capillary-force-induced nanoparticle aggregation processes, highlighting the importance of the interactions between the nanoparticles and the confining mineral walls. Our novel SFA-based approach offers insight into nano- and microscale mechanisms of consolidating silica treatments, and it can aid the design of nanomaterials used in stone consolidation.

## Introduction

**Figure 1:**
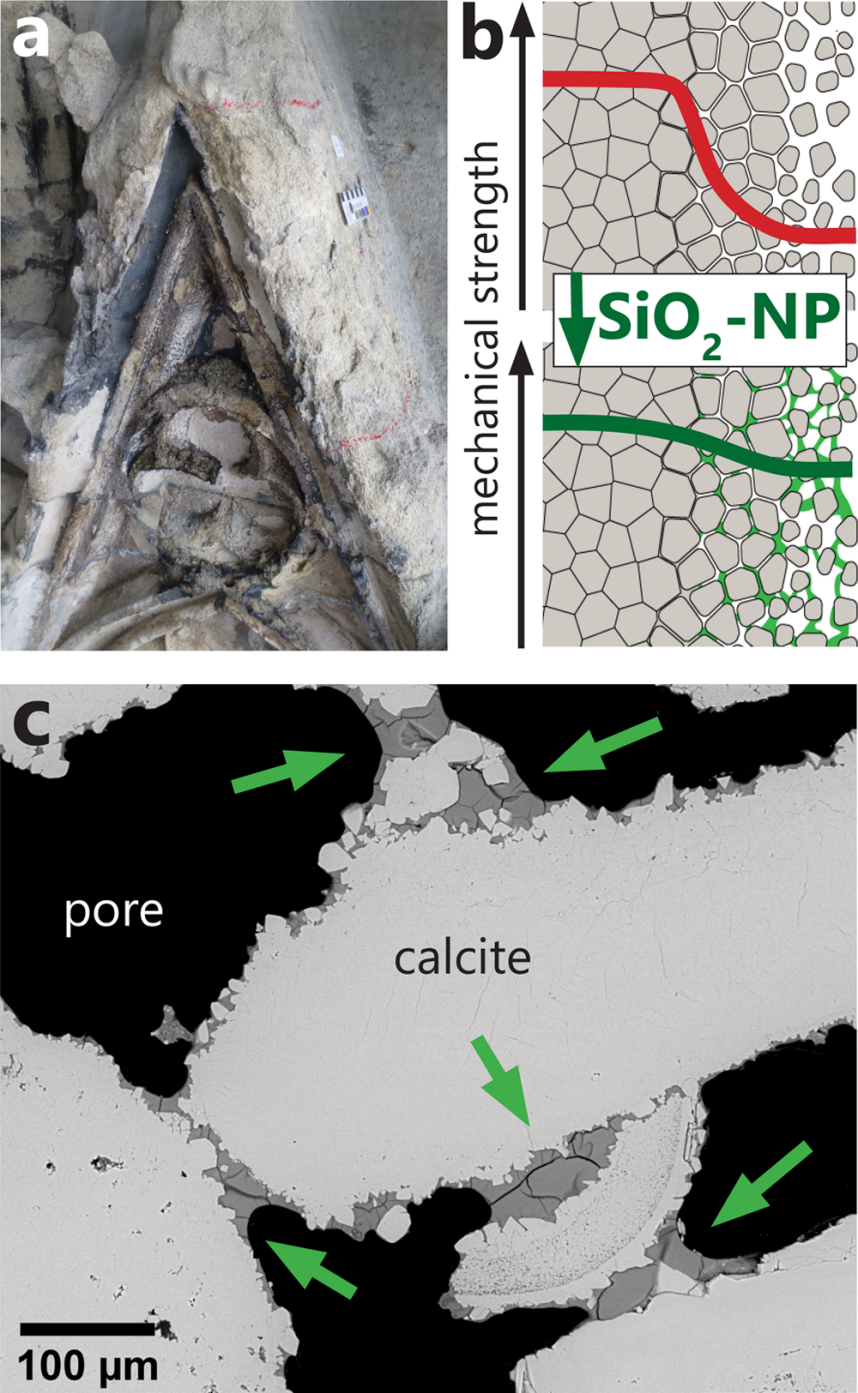
(a) Architectural element carved in St. Margarethen calcareous arenite on the facade of Vienna’s St. Stephens cathedral in Austria, exhibiting disintegration of rain-exposed surfaces. Weathering of such surfaces can be partially remediated with consolidant treatments. (b) A schematical stone degradation profile with the sketched mechanical strength decreasing from the bulk toward the weathered surface. Restoration of the lost cohesion between the grains can be achieved by applying stone consolidants such as silica nanoparticle suspensions (SiO_2_-NP). (c) Calcareous arenite rock sample (as in panel a) after the treatment with silica nanoparticles (SEM micrograph of a polished cross section). The distribution of cured nanosilica and its consolidating effect on individual calcite grains are indicated with green arrows. Black regions are preserved pore spaces. Panels a and c were adapted with permission from ref (4).

The aging of architectural stone surfaces exposed to the outside environment is inevitable. Porous building stones slowly decay because of chemical and mechanical weathering, which often progress together due to percolating rainwater, freezing and thawing, and temperature and humidity changes.^1,2^ These factors induce gradual stone deterioration, usually revealed in granular disintegration, porosity increase, mineral crystallization, crusting, salt efflorescence, and microcracking.^1,3^ When the cohesion between grains in a stone is lost, the mechanical strength decreases, and the physical properties of the building material change. Although such decay and loss of cohesion between grains are most severe at the exposed stone surface, the adverse effects frequently continue deeper into the bulk material (Figure 1a,b). Partial restoration of the stone’s mechanical and physical characteristics is, however, often possible with consolidant treatments^4^ (Figure 1b,c). Materials used for consolidation comprise various solvent-dispersed binding agents introduced into degraded surface layers of stone materials to restore the lost cohesion between mineral grains.^3^

Reconsolidation of disintegrated architectural surfaces remains challenging because of their complex microstructures and thus variation in pore sizes, microcrack apertures, and local chemical composition. Thus, consolidant suspensions often display stone-dependent efficiency, making it difficult to design more universal consolidating treatments. These treatments should possess good wetting properties for a wide range of mineral phases and low viscosity to enable their penetration into micrometer-sized cavities. Various nanomineral- and polymer-based consolidants have been demonstrated to partially restore the lost cohesion within the weathered stone surface layers. These most often include water or organic solvent-dispersed inert nanosized inorganic particles, which provide cohesion upon their aggregation (such as colloidal silica, calcium hydroxide, calcite, or metal oxides^5−9^) or synthetic organic polymers and reactive alkoxysilanes, which consolidate grains via self-polymerization reactions.^1,6,10^ Although grouting materials such as cement or organic adhesives may act as suitable cohesives and sealants in geotechnics,^11^ they are often inappropriate for cultural heritage applications as they may fail to preserve the initial surface appearance, lack chemical or microstructural compatibility with stone material, or induce undesirably drastic changes in the mechanical properties of the reconsolidated surface layers.

Here, we focus on water-dispersed, nanosized colloidal silica SiO_2_. The ability of silica nanoparticles (SiO_2_-NPs) to provide cohesion upon their aggregation is what makes them usable as consolidants improving the mechanical strength of porous materials,^12,13^ grouts and sealants that limit water infiltration into microcracks and cavities,^11^ protective surface coatings,^14^ or even adhesives used in deep wound healing.^15^

An effective nanoparticle-based consolidant should remain well-dispersed in a solvent phase and aggregate only upon the solvent removal. Aggregation of negatively charged colloidal silica particles (usually smaller than 100 nm in diameter) in aqueous solutions is generally governed by a balance between DLVO forces comprising electrical double layer and van der Waals surface forces. Thus, silica aggregation can be induced by changing the solution pH (which affects the surface charge of particles) and/or ionic strength conditions (which modify the range and magnitude of electrical double layer forces).^16^ However, because of the formation of nanometer-thin, hairy, polymeric gel layers on silica surfaces (especially present when the proportion of hydrophilic silanol −Si–OH groups on the surface is higher than of more inert siloxane units Si–O–Si), the silica aggregation behavior commonly shows deviations from the DLVO theory. In general, such gel enhances the colloidal stability of silica as it contributes additional short-range steric repulsive forces between SiO_2_ particles.^17,18^

Whereas the aggregation of SiO_2_ particles in aqueous solutions requires overcoming of repulsive double layer forces,^16−18^ the aggregation induced by drying is triggered by capillary pressure.^19^ When colloidal suspensions of silica dry on a solid substrate at ambient temperature and humidity conditions, the solvent slowly evaporates, leading to continuous densification and shrinkage of the drying matrix.^20^ At low solvent contents, the solution is only retained in the interparticle spaces in a form of menisci sustained by capillary forces. The attractive capillary forces push the particles into contact against repulsive colloidal forces,^21^ leading to silica aggregation.^22,23^ Such capillary-force-induced silica aggregation can provide substantial mechanical strength to the drying porous nanosilica matrix, even at a rather high relative humidity (RH).^21^ Ultimately, when the solvent fully evaporates, the silica may undergo sintering due to the formation of covalent siloxane bonds between the aggregated particles.^24,25^ However, in general, capillary bridges can persist between silica particles for extended times, especially at ambient temperature and humidity conditions.^26,27^ As such, the effect of the long-range attractive capillary forces in providing mechanical strength to the drying nanoparticle suspensions is usually larger than that of the other relevant short-range interactions such as van der Waals attraction or chemical siloxane bonding at particle contacts.^28^

However, in conservation science, because of significant microstructural and chemical heterogeneities of common building stones, the gain in mechanical strength after their reconsolidation is assessed at the macroscopic scale. Typical experiments focus on post-treatment changes in flexural or compressive strengths, elastic properties (e.g., Young’s modulus), or sound speed propagation.^29^ Although these procedures accurately verify the efficiency of a given consolidant treatment, they do not offer real-time nanometer- and micrometer-scale information about the consolidating mechanisms in action.

Here, we present a facile and robust method to study the strength of solvent-dispersed consolidants at single mineral contacts quantitatively using in-house-modified surface forces apparatus (SFA) with real-time force sensing.^30^ Complementary structural information about the nanosilica suspensions during drying, aggregation, and solidification is obtained from X-SFA experiments.^31^ Force sensing introduced in SFA allows measuring of high adhesion induced by consolidants confined between two mineral surfaces. This large-scale confinement is generally absent in atomic force microscopy (AFM), which makes the SFA superior in studying processes occurring at highly confined mineral grain contacts. Our experiments provide nanoscale real-time insight into the full consolidation process, including the interactions of the consolidating nanoparticles with the confining mineral grains. The proposed method can be easily expanded to test the effects of mineral grain surface properties such as roughness and wettability or various aggregation-modifying additives, offering new perspectives for the systematic development of more efficient consolidating treatments.^32^ In addition, as mica surfaces (used in this work as confining mineral grains) cannot represent the physicochemical properties of all commonly used monumental stones, our method can be easily extended to other mineral surfaces.^17,33^

## Materials and Methods

### Aqueous Nanoparticle Suspensions

We used commercially available colloidal silica nanoparticle (NP) suspensions (Ludox SM, Ludox HS, and Ludox TM) supplied by Sigma-Aldrich, Germany. The diameter of NPs was determined by transmission electron microscopy (TEM; FEI CM20 microscope) as 10.3 ± 2.0 nm (Ludox SM), 15.8 ± 2.9 nm (Ludox HS), and 26.1 ± 4.0 nm (Ludox TM). Prior to use, the NP suspensions were purified by dialysis using SnakeSkin tubes (3.5K MWCO, ThermoFisher, Germany) in Milli-Q water (18.2 MΩ·cm) for 10 days (with daily water exchange). Concentrations of the dialyzed suspensions were characterized by weighting the samples before and after drying (24 h at 80 °C in a vacuum). The suspensions densities were determined by weighing the water-suspended and dialyzed NPs with known concentrations by using a high-precision volumetric flask. The density for silica was determined to be 1.97 ± 0.07 g cm^–3^. The suspensions were further diluted with Milli-Q water to achieve desired concentrations varying between 6 and 16 wt %. We additionally used a colloidal silica of 20 ± 3 wt % suspended in a 50:50 water–ethanol mixture by volume. The product (ZG12) was developed by Colorobbia Italia S.p.A. (Sovigliana Vinci, Firenze, Italy). The mean diameter of the NPs was 70 ± 30 nm as analyzed with DLS (Brookhaven Instruments, USA). The density of the product at 25 °C was 1 g mL^–1^, and the viscosity was 2 mPa·s. If needed, the 70 nm silica suspension was diluted keeping the water-to-ethanol ratio.

### Surfaces

We performed all SFA experiments using two thin (<10 μm), freshly cleaved, optical-grade, and atomically smooth single-crystal muscovite mica surfaces glued onto the standard SFA cylindrical disks with a 2 cm radius of curvature. To facilitate the interferometric analysis, the micas were back-coated with a thin, 40 nm layer of Ag or Au with an in-house-built thermal evaporator to yield transparent, semireflective surfaces, which allow the collection of fringes of equal chromatic order (FECO) in the spectrometer. In each experiment, the two opposing mica surfaces were crossed at 90° to yield a spherical contact area (see standard SFA mica preparation details in refs (34 and 35)).

### Surface Forces Apparatus with Real-Time Force Measurements

Breaking, shearing, and normal forces were measured with the in-house-modified surface forces apparatus (SFA), equipped with two strain gauge-type force sensors (ME-Meßsysteme GmbH; coupled with the GSV8 controller), detecting forces (>1 μN) in the normal and shearing directions in real time. The detailed design of the apparatus is presented in Wieser et al.^30^ Owing to the high-resolution and distance-independent force signal (now decoupled from the interferometric surface separation measurement unlike in traditional SFA and surface force balance (SFB) experiments^36^), large adhesive forces can be precisely and quickly measured even at very high surface separation velocities, >1 μm s^–1^. Traditional data analysis of such strongly adhesive forces would require tracing of the interferometric fringes of equal chromatic order (FECO) after the adhesive jump-out events (to hundreds of micrometers), back to the contact position at zero separation, leading to a time-consuming adhesion force calculations. Moreover, the modified SFA facilitates the use of any nontransparent surfaces for any high adhesion force experiments because the FECO-calculated separation distance is no longer needed to calibrate the measured forces.

### Breaking, Shearing, and Normal Force Experiments

Breaking experiments were performed by pipetting a 1 μL droplet of a given silica suspension onto the very center of the bottom mica surface. The volume of silica suspension was strictly controlled to compare the magnitude of cohesion. We used a precise micropipet to ensure reproducible droplet volumes. Quickly after, the top mica surface was mounted rapidly onto the SFA holder, and the surfaces were manually brought into contact. Once the surfaces were almost in contact, a capillary bridge of silica suspension formed between the surfaces. We further approached the surfaces until the contact was established (as indicated by the instantaneous increase in the measured normal force). The final moderate load, applied throughout the whole duration of breaking experiments, was adjusted with a piezocontroller operated with the LabVIEW code. The drying nanosilica suspension gradually pushed the surfaces toward each other, and once dried (as indicated by cracking of solidified silica glass visible in top view SFA optical camera), it kept them in a highly adhesive contact. We then separated the surfaces at very high velocities (>1 μm s^–1^) using the piezocontroller to break the formed nanosilica bridges. The resulting highly adhesive force (“breaking force”) was normalized with the contact radius of curvature determined from the shape of interferometric fringes (with mica surfaces kept out of contact). For the smallest NP (Ludox SM), the breaking force was so high that we could not separate the mica surfaces by moving the surfaces within the whole piezocontroller range (∼100 μm). In these cases, a droplet volume was decreased to 0.5 μL, and the adhesion breaking force was normalized with respect to the used volume to allow the comparison between experiments with 1 and 0.5 μL droplet volumes. Mica surfaces were usually reused for a few breaking experiments. If so, the surfaces were sonicated in and rinsed excessively with Milli-Q water and then dried under N_2_ stream after each run. We have not observed any significant damage on the reused mica surfaces with the SFA top camera, and the contact area was located in a different place when reassembling the surfaces. The breaking experiments were performed at a temperature of ∼25 °C and a relative humidity of ∼50%.

Shearing force experiments were performed with 2–5 μL nanosilica suspension droplets injected between two opposing mica surfaces. Here, the droplet volume was not strictly controlled as we only investigated evolution of surface forces during the drying process. At the start of each experiment, the surfaces in crossed-cylindrical configuration were precisely aligned by using two independent goniometers until the distance between the surfaces was approximately constant while shearing over micrometer-scale lateral distances, as monitored by using the FECO interferometric fringes. The surfaces were slided past each other until the nanosilica droplet became dry. The shear force signal was recorded throughout the whole drying experiment.

We additionally measured normal forces between two mica surfaces, fully immersed in nanosilica suspensions, as in standard SFA experiments in liquids. The normal forces acting between mica surfaces were recorded as a function of surface separation to monitor possible aggregation of nanosilica particles onto mica in wet conditions. The used liquid cell had a volume of 5 mL, and no substantial solution evaporation occurred within the time scale of the experiments (1–2 h).

### X-ray Scattering Experiments

Complementary information about the silica droplet drying process was simultaneously monitored in a surface forces apparatus specially adapted for *in situ* X-ray scattering experiments (X-SFA). X-SFA shearing experiments provided structural information from the drying SiO_2_-NP suspension inside a slit-shaped SFA pore (here formed between a mica surface glued onto cylindrical SFA disk with a radius of curvature 1 cm and a flat gold template-stripped substrate to provide a more suitable geometry for the *in situ* X-ray measurements). While shearing the suspension, the slit pore was gradually closed to ∼900 nm. Humidity in the X-SFA chamber was decreased in the course of experiments to induce droplet drying (see humidity conditions in Figure S3). The X-SFA experiments were performed at the Swedish Materials Science Beamline P21 at the Petra III synchrotron, DESY, Hamburg. A microfocused X-ray beam (1.2 μm vertical, 5 μm horizontal; X-ray energy 69.5 keV) penetrated the pore in the direction of the top surface cylinder apex. The detailed design of the X-ray-adapted SFA and the experimental methodology are more thoroughly described in Weiss et al.^31^

## Results and Discussion

Proper consolidating action of nanoparticles within a porous stone matrix requires their twofold behavior: initially, the solvent-dispersed nanoparticles should interact poorly with the mineral matrix while injected into stone’s pore space to allow proper penetration depths and to limit possible pore clogging; however, at later stages, when the solvent evaporates, the nanoparticles should adhere strongly to the solid matrix walls and to each other to provide lasting cohesion. We thus first investigated how the water-dispersed nanosilica particles bind to smooth mica surfaces, which comprise confining mineral walls in our model system.

**Figure 2:**
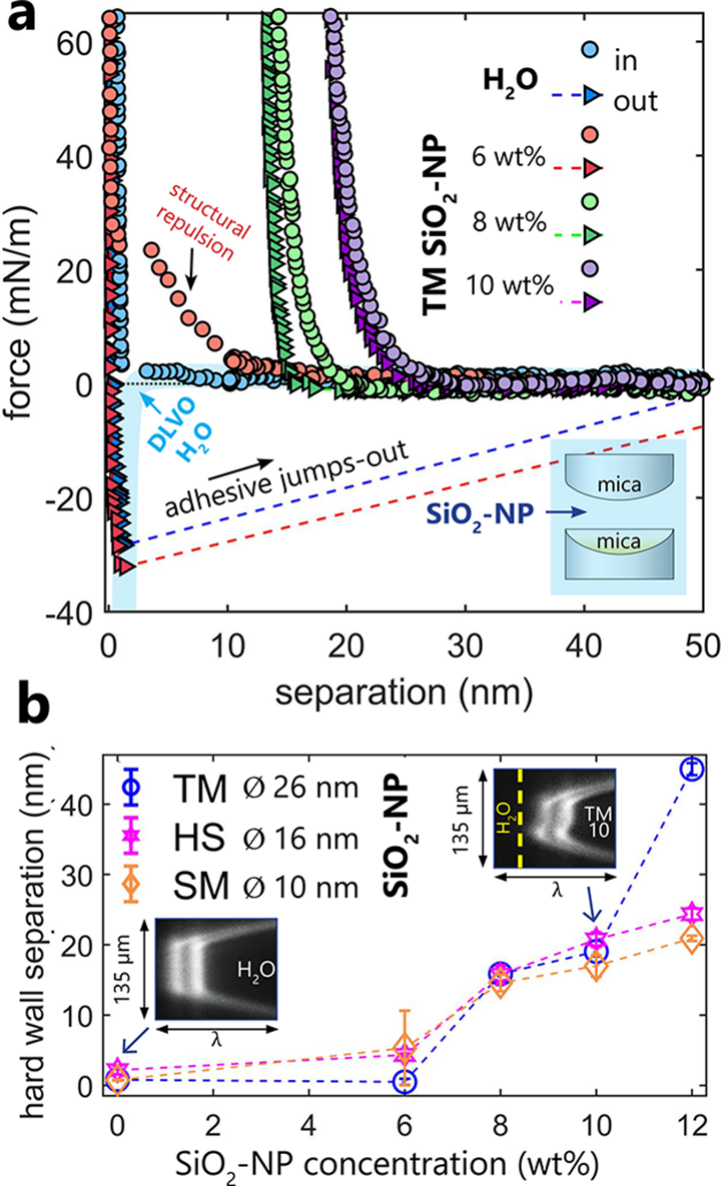
(a) Forces measured between two mica surfaces, fully immersed in Ludox TM SiO_2_-NP suspensions with different silica concentrations, or in Milli-Q water (H_2_O). We first measured forces in H_2_O and subsequently injected silica suspensions in the order of their increasing concentration. All forces were measured in the same contact area. A DLVO fit for H_2_O is shown for reference (see the fitting parameters in the Supporting Information). (b) Shift of hard wall position as a function of initial concentration of nanosilica suspensions for Ludox TM, HS, and SM SiO_2_ nanoparticles extracted from the force measurements between two mica surfaces shown in (a). The insets show FECO fringes corresponding to the measured hard wall contact separations for mica surfaces in water or in 10 wt % Ludox TM suspension (the yellow dashed line marks the initial hard wall position in water). The observed shift in the FECO position corresponds to deposition of silica onto mica surfaces.

Figure 2a shows standard SFA force measurements between two mica surfaces, fully immersed in colloidal silica nanoparticle suspensions (SiO_2_-NP) with a varying initial silica concentration. For each pair of mica surfaces, we first measured reference forces in pure Milli-Q water (ionic strength of ∼5 × 10^–5^ M; ref (37)). As expected, we observed weak, longer-ranged electrical double layer (EDL) repulsion (Debye length, κ^–1^ = 43 nm) dominated by van der Waals (vdW) attractive forces at smaller separations, as evident from a small jump-in on approach and a larger adhesive jump-out on retraction.^34^ The expected magnitude of these DLVO^16^ forces in water is sketched in Figure 2a (see the DLVO fitting parameters in the Supporting Information). We then injected SiO_2_-NP, in the order of increasing NP concentration. Adhesion was still preserved at the lowest 6 wt % concentration for all NP sizes, as shown in Figure 2a for the TM SiO_2_ particles (ϕ ∼ 26 nm) and in Figure S1 for the other particle sizes. Here for TM NPs, in contrast to the forces measured in water, a significant non-DLVO exponential repulsive force during the approach appeared before the attractive jump-in event. We attribute this repulsion to the progressive removal of silica nanoparticles from between two mica surfaces upon the increasing confinement. This structural repulsion is in agreement with the oscillatory depletion force originating from a layerwise expulsion of nanoparticles from the contact region, which was previously observed across confined nanoparticle suspensions in colloidal-probe AFM.^38,39^ Because of the different geometry and sensitivity of our SFA setup, we do not resolve such oscillations, which are detectable below 1 mN/m. In the case of the lowest concentration (6 wt %) of the largest Ludox TM particles, we did not observe any shift of the hard wall contact position with respect to that measured in pure water. This points to the complete removal of silica NPs from the confined zone upon loading and no NP adsorption onto mica within the contact region between two mica surfaces (Figure 2a,b). The existence of this NP-free depletion layer in the confined region between two mica surfaces (in agreement with the electrostatic repulsion between negatively charged silica and mica) may enhance the attractive forces between micas across NP suspensions with respect to pure water due to the depletion attraction effect.^38^ Smaller Ludox HS (ϕ ∼ 16 nm) and SM (ϕ ∼ 10 nm) particles already showed a minor shift in hard wall position of <5 nm even at the lowest 6 wt % concentration, which suggests their very minor adsorption on mica. In these force measurements with the hard wall shift present, the depletion force was not detected. Note that in SFA the shift of the hard wall may appear smaller than the average particle diameter if the surface density of particles is below the lateral resolution of SFA (i.e., the average hard wall position between particle covered and clean areas is measured).

At higher particle concentrations, we observed more significant shifts in hard wall contact separation. Figure 2b shows that the maximum shift in hard wall position at SiO_2_-NP concentrations between 8 and 12 wt % was generally in the range of ∼20 nm for different silica NPs (apart from 12 wt % Ludox TM with a 45 nm shift). This, along with the contact shape outlined by the interferometric FECO fringes^40^ (see FECO insets in Figure 2b), indicates that the silica nanoparticles did not adhere uniformly onto mica but adsorbed in low quantities and in a discontinuous fashion. The total shift of ∼20 nm (for two opposing mica surfaces together) shows that the adsorbed NPs rarely exceeded a monolayer thickness on a single mica surface within the contact region. Such discontinuous silica adsorption on mica gave rise to similar hard wall positions measured for silica NPs with different sizes, especially at their lower concentrations. In addition, above 8 wt % concentration of SiO_2_-NPs, we now only measured purely repulsive forces both on approach and on retraction for all NPs sizes, as shown for Ludox TM NPs in Figure 2a. This indicates that the thin and discontinuous layers of SiO_2_-NPs that deposited onto micas prevented the mica surfaces from reaching adhesive contacts at moderate applied loads (<60 mN m^–1^). We used a decay length parameter (λ) to account for the magnitude and range of the measured exponentially decaying steric repulsion, as shown in Figure S1. A lack of clear trend in λ as a function of SiO_2_-NP size and concentration points to two main distinct origins of the repulsive forces: At lower 6 wt % SiO_2_-NP concentrations, where the surfaces could approach each other to small surface separations below 10 nm, the repulsion was dominated by structural depletion forces. At higher concentrations, the adsorbed particles could not be displaced from the contact region any longer at the applied load range that we used. Thus, the measured repulsion was mainly related to the roughness of now nanoparticle-laden mica surfaces and had its origin in mechanical deformation of protruding nanoparticle asperities upon their compression.^41^ Variation in λ at higher SiO_2_-NP concentrations can be related to a nonuniform deposition of NPs in the contact region, as only locally deformed asperities (composed of adsorbed NPs) may contribute to the measured repulsion.^33^ In contrast, in colloidal probe experiments, there is no hint for a shift in hard wall contact separation, even at 10 wt %.^39^ A steep repulsion has been obtained at short separations. Reasons for the difference might be a slower approach speed (100 nm/s) and a smaller contact area (∼μm) than in SFA experiments. In addition, differences in the physicochemical characteristics of the confining surfaces (colloidal silica in AFM and mica in SFA) may contribute to the different behavior of these two systems.

On the basis of these observations, we infer that the water-dispersed negatively charged SiO_2_-NP do not deposit spontaneously onto mica from the aqueous phase but are rather pushed onto the confining mica surfaces during the repeated loading–unloading cycles in SFA. The externally applied load forces SiO_2_-NP to contact mica at surface separations where the adhesive vdW forces overcome the electrostatic repulsion between like-charged silica and mica, trapping some of the silica particles onto the mica surfaces (the majority of the NPs are, however, still depleted from the confined zone due to electrostatic repulsion). Such forced deposition of silica onto mica is not reversible on exchanging the solution once the silica particles are pushed into the adhesive vdW minimum. In relation to the consolidant treatments, where no applied load is present, the observed repulsion between the nanoparticulate water-based consolidant and the mineral walls may aid the good spreading of the consolidant within the pores of degraded stone material.

**Figure 3:**
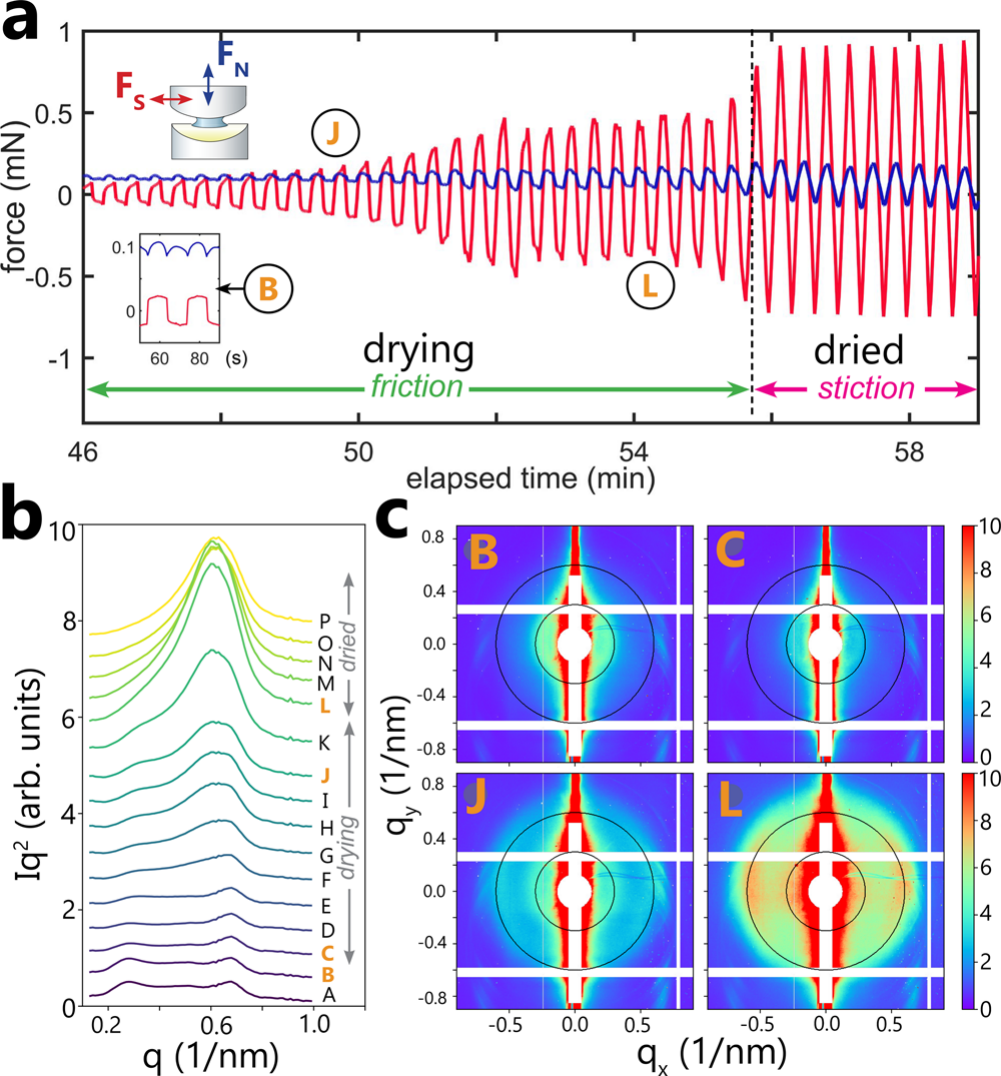
(a) Shear force (*F*_S_; red) and normal force (*F*_N_; blue) measured in the SFA during drying of a Ludox SM (ϕ = 10 nm) 6 wt % suspension droplet as a function of time elapsed from the droplet injection. The full shearing pattern is shown in Figure S2. Once the silica droplet dried (at relative humidity, RH = 0.5), the shearing force significantly increased and evolved from a typical friction pattern (with the magnitude of *F*_S_ measured during the initial seconds of the shearing as plotted in the small inset) into stiction (due to silica bridges providing very strong cohesion to the confining mica surfaces). The letters correspond to a typical X-ray signal measured at different stages of the drying process. (b) *In situ* X-ray scattering patterns of 12 wt % Ludox SM suspension confined in a slit-pore X-SFA geometry measured during silica droplet drying and simultaneous shearing at 900 nm gap width. The A-P X-ray signal lines correspond to the radial averaged and normalized intensity *Iq*^2^ vs *q* at ±30° azimuth relative to the horizontal direction: (A–B) silica suspension after injection at 100% RH.; (C–J) progressive drying of suspension for 50 min with the humidity dropping to 30% (the gradual shift of the peak at 0.3 nm^–1^ indicates increasing NP concentration); (K–P) final dry-out of the contact with a rapid increase of shear forces and strength of the scattering signal. (c) Representative 2D scattering patterns. The feather-like features around 0.7 nm^–1^ originate from scattering at the confining substrates. The concentric black circles indicate a momentum transfer *q* = 0.3 nm^–1^ (NP suspension) and *q* = 0.6 nm^–1^ (amorphous precipitate).

We subsequently investigated forces acting within a small-volume, drying nanosilca consolidant droplet, trapped between two opposing mica surfaces by capillary forces. As such, we were able to monitor interactions between mica surfaces across the formed capillary bridge and between NPs themselves during water evaporation and later NP aggregation. To obtain complementary information about these forces and the microstructural evolution within the drying silica suspensions, we combined friction (*F*_S_) and normal force measurements (*F*_N_) with X-ray scattering using the X-SFA setup^31^ as shown in Figure 3. During the collection of the X-ray signal, the mica surfaces were constantly sheared against each other. The measurement of frictional forces allowed us to monitor the interactions within the droplet without a change in mica surface separation, which facilitated the simultaneous X-ray structural data collection.

Figure 3a shows the resultant friction force (*F*_S_) trace recorded on drying. For the first ∼45 min of the drying process, we did not observe any significant increase in *F*_S_ or *F*_N_, as shown in the full friction trace in Figure S2a,b. Such low frictional forces correspond to a contact that remains well lubricated by the silica colloidal suspension. However, in the last ∼10 min of the drying, we observed a significant increase in the friction force signal, with a maximum in *F*_S_ corresponding to dried silica nanoparticles bridging two mica surfaces. As such, the force evolved from a typical friction pattern observed for silica particles suspended in water to a stiction pattern, which indicates strong adhesion between mica surfaces. Such strong stiction shows that the dried, aggregated silica particles bound the two mica surfaces together. The formed silica bridges could withstand shear stress due to the lateral movement of two mica surfaces (with a total amplitude of ∼10 μm) without breaking.

The final drying stage of silica suspension zoomed-in Figure 3a could be characterized by a high friction coefficient, μ ∼ 8.5 (as determined assuming Amontons’ friction law:^42^; see Figure SI2c). This high μ, observed at the final stages of drying, revealed a significant viscosity increase and the increasing resistance to the imposed shear in the drying and densifying silica droplet. However, the final appearance of the stiction regime for dried silica can be related to the silica aggregation induced by capillary interparticle forces.

In our drying experiments, the capillary force acts in two ways. First, as the capillary bridge formed by a SiO_2_-NP droplet between two mica surfaces shrinks during solvent evaporation, the increasingly negative capillary pressure (*P*_c_) pushes the top mica surface toward the bottom one (the top surface is not rigidly fixed in our SFA and X-SFA setups). This is related to the decreasing radius of a capillary bridge meniscus (*r*_M_)^43^ formed by the silica suspension as indicated by eq 1,^26^ where γ is water–air interfacial tension:
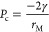
1This phenomenon causes the normal force load *F*_N_ to increase with time during the droplet evaporation, as plotted in Figure 3a. Second, at later stages, when enough liquid has evaporated and silica solidifies, capillary forces start to act also across liquid bridges maintained between individual silica nanoparticles, leading to their aggregation. Aggregated silica nanoparticles provide cohesion to the two confining mica surfaces and they are strongly cohesive themselves, leading to the stiction observed at the final stages of the experiment. The increase in *F*_S_ could be also in a minor part attributed to the depletion of silica nanoparticles in the very contact area, which may have enhanced the adhesion between the mica surfaces.

The discussed changes in friction pattern agree well with the microstructural information accessed simultaneously with the X-ray scattering shown in Figure 3b,c. In a wet state, the recorded X-ray patterns from the confined SiO_2_-NP colloidal suspensions resembled a diffuse halo at ∼0.3 nm^–1^ (Figure 3b: A–B). This signal originated from the interparticle scattering of stable SiO_2_-NP suspensions with a mean real space periodicity of 20 nm, in agreement with SAXS measurements by Zeng et al.^44^ Vertical streaks and feather-like features at 0.7 nm^–1^ are caused by X-rays reflecting at the solid–liquid interfaces. An additional scattering peak emerged after we initiated the dry-out process of a silica NPs droplet by decreasing the humidity in the X-SFA chamber (see Figure S3). This second peak at larger scattering angles of 0.6 nm^–1^ (that appeared after ∼20 min of drying) indicates that the interparticle distances decreased and amorphous precipitate began to be formed. This larger scattering angle corresponds to a real space periodicity of ∼10 nm, in agreement with the diameter of Ludox SM NPs determined by TEM. The gradually increasing relative intensity of the second peak (Figure 3b: F–J) reflected the increasing fraction of the dried aggregated NPs. After ∼50 min, the first peak has disappeared (Figure 3b: N), indicating that the silica droplet dried completely. The significant changes in the X-ray scattering after ∼45 min (Figure 3b: K) mark the transition correlated to a strong increase in the simultaneously measured shear forces and indicate nearly complete evaporation of water from the NPs suspension. As such, the X-ray-detected silica solidification was correlated to the highest measured friction force (see Figure S3). Interestingly, in all our X-SFA experiments we only observed amorphous scattering signals from the silica NPs. No indications for crystalline NP aggregates were found (although these has been reported to form with Ludox SiO_2_-NPs drying on isolated substrates^45^). Furthermore, apart from shadowing and reflection effects from the solid substrates, no anisotropy of the scattering patterns could be detected. Thus, our data indicate the formation of isotropic silica glass. It is however important to note here that the microstructures of the dried silica glass could have been to some extent affected by the presence of shear and may differ from microstructures obtained in the absence of shear.

**Figure 4:**
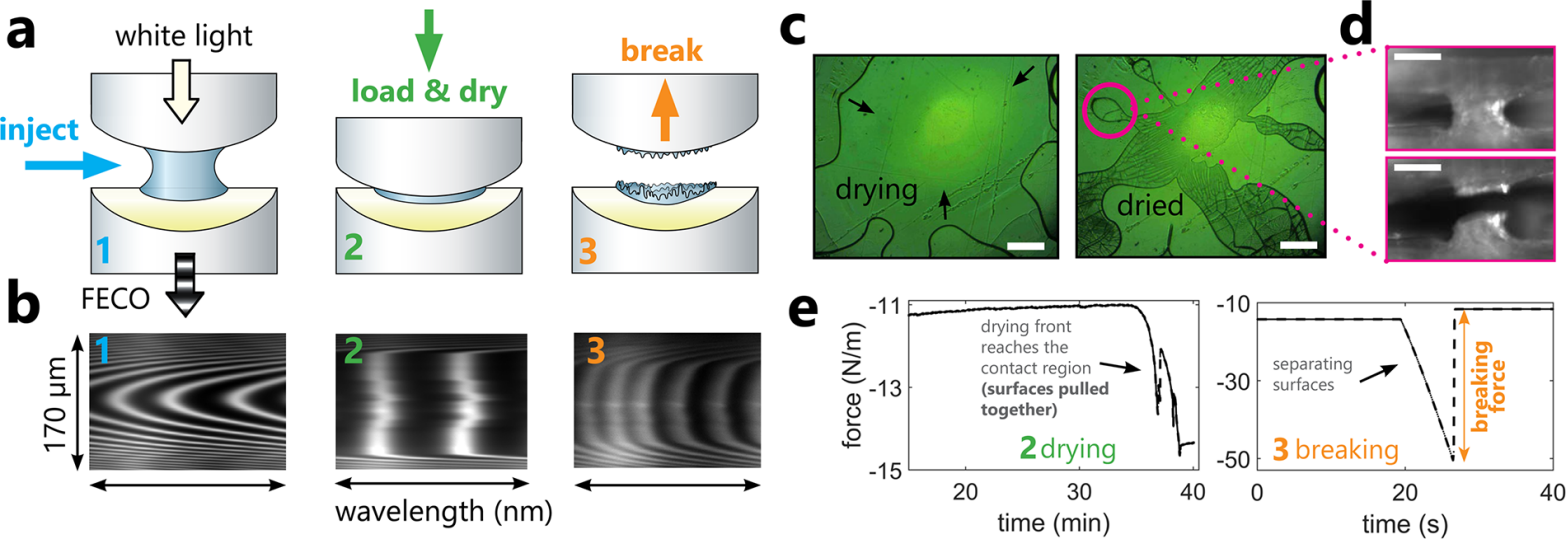
Schematic representation of the breaking force experiments in the SFA. (a) Crossed-cylindrical configuration of the surfaces with three experimental steps: droplet injection, loading and drying, and breaking. The white light passes through the two semitransparent metal-coated mica surfaces at all times, producing interferometric fringes of equal chromatic order (FECO). These FECO fringes provide information about surface separation across the whole contact region with a diameter of approximately 150–200 μm. (b) FECO patterns corresponding to droplet injection (surfaces out of contact), loading and drying (surfaces are contacted at moderate load and then pulled together to form a large flat contact in the course of drying), and breaking (the surfaces jump out to very large separations during the breaking event). (c) Top SFA camera view on one of the mica surfaces showing a drying nanosilica suspension droplet, moving toward the contact region between two mica surfaces (left) and the fully dried droplet (at RH = 0.5) with a characteristic crack pattern (right). (d) Side SFA camera view on one of the dried silica bridges before (top) and after (bottom) the breaking event. The shown silica bridge breaks without getting disattached from any of the mica surfaces. The scale bars in (c) and (d) are 0.1 mm. (e) The corresponding strain gauge force signal measured during drying (the surfaces are strongly pulled together once the drying front reaches the contact region) and breaking (surfaces are separated at a high velocity >1 μm s^–1^, as visible in a linear decrease in force, until the sudden breakage of the dried silica bridges occurs).

Given the high cohesion provided to two opposing mica surfaces by the dried and aggregated, glass-forming, amorphous silica nanoparticles, we then measured the mechanical (tensile) strength of dried silica bridges by performing “breaking force” experiments. Figure 4a shows a schematic representation of our SFA setup used to measure the breaking force (Figure 4e). As in friction force experiments, the injected droplet of nanosilica suspension is held between two mica surfaces mounted in crossed-cylindrical geometry by capillary forces. The droplet dries, undergoes densification, and shrinks, pulling the two surfaces together (see FECO interferometric, topography-sensitive pattern of a dried flattened contact between two mica surfaces in Figure 4b and force signal showing a sudden decrease in the measured force in Figure 4e). During droplet evaporation, the drying front moves toward the center of the contact between the mica surfaces, concentrating the majority of the silica NPs around the contact in a form of disconnected islands that bridge two mica surfaces together (see Figure 4c and Figure S4). The irregular bridges formed by dried SiO_2_ concentrate around the contact region. Because of the applied load and the additional capillary pull, there is very little material in the very center of the spherical contact region (of ≈100 μm, where the distance between the surfaces is the smallest; less than a few nanometers). Despite extensive cracking upon drying,^26^ the dried silica nanoparticles form very strong bridges that keep the mica surfaces together (Figure 4d). We then break these bridges by separating the consolidated mica surfaces at a high constant velocity. This tensile test causes the force signal to decrease linearly until the breaking event occurs: the surfaces suddenly separate, and the force immediately jumps back to the initial level (Figure 4e). The difference between the minimum measured force before the breaking event and the force level after breaking lets us quantify the consolidation strength (in a uniaxial tensile test) termed the “breaking force”.

**Figure 5:**
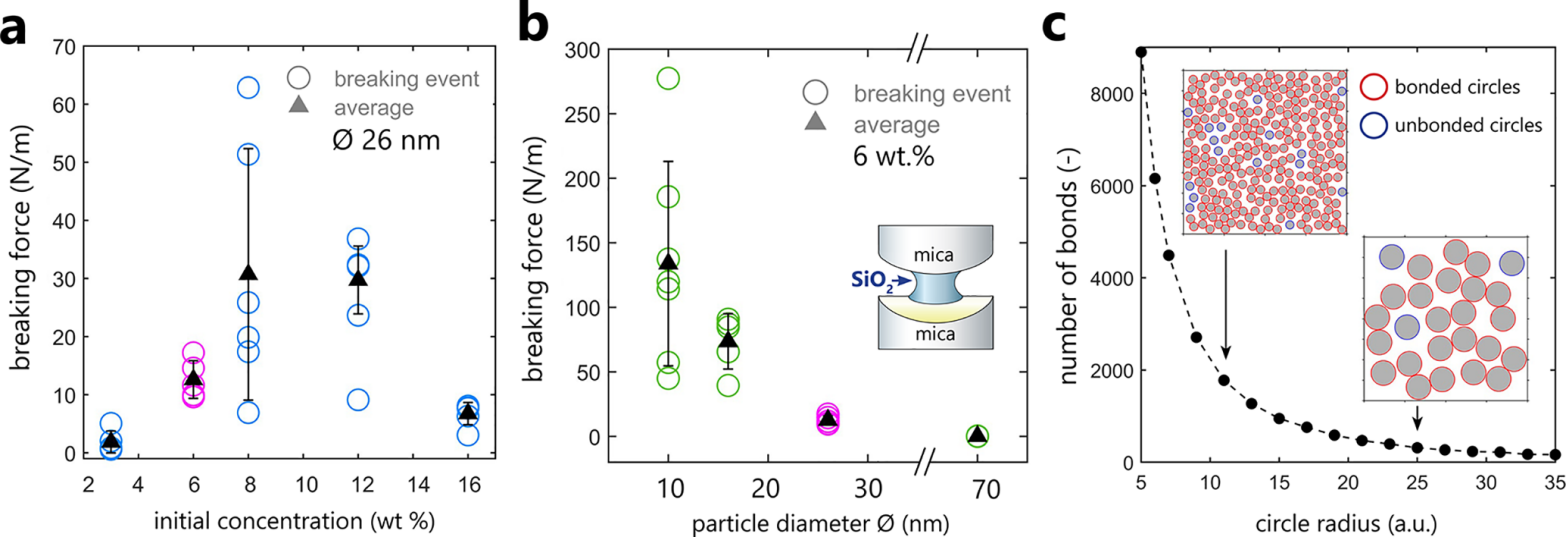
Breaking force measurements as a function of (a) silica particle diameter ϕ at a fixed initial concentration of 6 wt % and (b) initial concentrations of nanosilica suspensions for SiO_2_ nanoparticles with ≈26 nm. The breaking force (N/m) was normalized with the radius of curvature of the cylindrical confining walls. The data plotted in magenta show experiments for the same particles with ≈26 nm and 6 wt % concentration. (c) Simplified 2D modeling of a number of cohesive bonds between randomly packed circles as a function of circle radius within a given fixed surface area.

Figure 5 shows the results of breaking experiments with breaking force studied as a function of initial silica nanoparticle concentration (panel a) or as a function of silica nanoparticle average diameter (panel b). Despite a quite complex arrangement of the irregular islands formed by the dried NPs around the contact region (see Figure S4), both parameters influenced the magnitude of the measured tensile strength of contacts in a reproducible manner. Figure 5a shows that the breaking force increased with the increasing initial concentration of silica nanoparticles, apart from the highest 16 wt % concentration. Such larger mechanical strength of contacts at higher SiO_2_ concentrations can be simply linked with a larger surface area of the dried silica bridge regions due to a higher amount of the SiO_2_-NP material. The change in this trend at the highest 16 wt % SiO_2_-NP concentration points to a possible change in the aggregation kinetics and the resultant silica bridge distribution: because of the higher NP density, silica may aggregate faster, leading to clustering and less material reaching the most confined regions. Thus, the bridging becomes less effective as the bridges have to be longer at higher surface separations. Faster aggregation kinetics has been previously demonstrated for concentrated silica nanoparticle suspensions.^46^ In addition, the drop in mechanical strength at the highest concentration may be related to changes in the packing fractions and coordination number of the nanoparticles within the bridge volume, yielding final bridge structures with lower densities. A decisive influence of the initial aggregation kinetics on the final microstructure of the dried silica has been underlined in recent self-assembly experiments of Ludox SiO_2_-NP on flat, unconfined surfaces by Lesaine et al.^45^

The size of nanosilica particles had a much more significant influence on the measured breaking force, with the smallest NPs yielding much stronger (by 1 order of magnitude) consolidated contacts than the largest ones as plotted in Figure 5b. The apparent dependence of the breaking force on the particle size (at the same initial SiO_2_ concentration) is related to the microstructure of nanoparticle aggregates and the cohesive forces acting between the individual NPs. For smaller nanoparticles, the coordination number of the nanoparticles is higher; there are more cohesive bonds between the particles in a given volume in comparison with the larger ones. We can illustrate this effect in a simplified way by considering a random packing of circles with a uniform radius in a given fixed 2D area. The 2D random packings of circles were generated by using an open-source MATLAB code.^47^ Assuming that the cohesive bonds between circles are only formed at distances between circles smaller than 0.5 of a circle radius, in Figure 5c, we plot how the number of cohesive bonds decreases exponentially with the increasing circle size in a square box with a fixed surface area. As such, with comparable packing fractions for all tested circle sizes (here of ∼0.5, which is in range for a random packing of monosized spheres with packing fractions varying between 0.56 and 0.64^48^), smaller particles can form many more cohesive bonds.

Although drying-induced self-assembly of silica nanoparticles has been demonstrated to be a complex and sensitive process, yielding crystalline or amorphous structures depending on small details in particle size, size polydispersity, or suspension drying rates,^45^ our measurements show robust and apparent dependence of the SiO_2_-NP mechanical strength on particle size and the initial concentration of SiO_2_-NP in the suspensions. This points to the origin of the tensile strength of granular aggregated solids, where the cohesive behavior of the bulk is governed by interparticle forces that can only be transmitted across contact regions between individual particles as proposed by Rumpf (1974) and described in refs (49 and 50). As such, the tensile strength (σ) of an aggregate composed of randomly packed, monosized, hard spheres scales inversely with the particle radius (*R*_p_), in the fashion corresponding to the number of cohesive bonds plotted previously in Figure 5c:
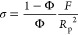
2where Φ is aggregate porosity and *F* is attractive interparticle force of a single particle–particle contact^49^ (see σ plotted as a function of *R* and Φ in Figure S5). This explains the robustness of cohesive nanoparticles in consolidating treatments where σ is less affected by slight variations in packing fractions and density of dried aggregates (induced by variations in size, size polydispersity, suspending medium conditions, drying rates, or relative humidity). Thus, based on our measurements, such a particle coordination effect appears to be more important than the higher magnitude of capillary forces or vdW for larger particles at a given surface separation. The dependence of these forces on particle size calculated for two spherical particles^16,51,52^ is plotted in Figure S6. Because vdW forces have a much smaller range than capillary forces and a weaker magnitude (especially in water) at surface separations smaller than nanometers (for nanosized particles), we assume that the mechanical strength in our system is provided mainly by capillary forces, in agreement with other works.^19,26,27,45,53−55^

We can investigate this further by estimating the mechanical tensile strength of the formed silica bridges. On the basis of a detailed SEM analysis of broken silica bridge surface areas common for the two opposing mica surfaces after the breaking event (as marked in green in Figure S4), we can quantify the tensile strength of a consolidated contact. Using the breaking force measured for the smallest SiO_2_ nanoparticles (Ludox SM6), we obtained a value of 0.55 MPa (corresponding to adhesion energy of ∼20 J m^–2^). Even for the smallest NPs that provide the largest breaking force, this is 1–2 orders of magnitude less than typical tensile strength (7–70 MPa) of ordinary glass with covalent Si–O bonds,^56^ suggesting that covalent bonds are probably not formed at silica contacts in our setup within the time scale of our experiments. Instead, we infer that the mechanical strength is mainly provided by capillary forces that act across the preserved liquid menisci.

Here, it is important to note that in all our breaking experiments we always observed two modes of consolidant bridge failure: the first which breaks off smoothly at one of the mica surface and the second which breaks across aggregated silica in the middle of a bridge yielding a rough surface (see uncolored and green areas in Figure S4 and Figure 4d). This means that both of these failure modes must have a quite comparable strength (otherwise we would observe only one type of failure). However, the proportion of the first type of bridges (breaking off at the mica surface) was in general slightly larger. This suggests that the mica–silica contacts are weaker and that more insight into possible ways of strengthening the consolidant–substrate adhesion in a dried state is needed.

Although muscovite mica used in this work as confining walls is convenient for SFA measurements, this aluminosilicate cannot represent all mineral surfaces commonly present in various monumental stones, especially those more reactive such as carbonates. Therefore, our work should be extended to other mineral surfaces comprising confining walls. This is feasible as minerals such as calcite or SiO_2_ have been previously used in SFA measurements.^17,33,57^ The interactions between confining surfaces and consolidant particles but also between the consolidant particles themselves may be also largely modified by their surface roughness characteristics. This is another parameter that requires a further insight as natural surfaces are rarely as smooth as optical grade micas typically used in SFA.

## Conclusions

Our newly developed SFA methodology, aided by X-SFA structural measurements, not only enables quantification of the tensile strength of nanoparticle-cured mineral contacts but also provides a systematic insight into the nano- and microscale details of the consolidation process at various stages of the consolidating treatment. We demonstrated that capillary force-induced cohesion induces a substantial gain in the contact’s mechanical strength that is reproducibly affected by the silica nanoparticle size and concentration. The cohesion gain reaches up to 1 order of magnitude for the smallest tested silica nanoparticles. The contact strengthening is directly related to particle coordination and thus to the number of adhesive bonds that each particle can form, explaining the most efficient consolidating action for the smallest nanoparticles. The overall cohesion gain in our setup could be further improved by enhancing poorer adhesion between silica nanoparticles and the confining mineral walls. The versatility of our SFA approach gives insight into a broad range of consolidating parameters and paves the way to tailor-made solutions for more efficient restoration of built cultural heritage. Our approach may also be relevant in many industrial and environmental applications based on nanoparticle aggregation processes. Because mica surfaces used in this work cannot represent the physicochemical properties of all commonly used monumental stones, our SFA setup should be further extended to include other confining mineral surfaces. Although silica nanoparticles displayed robust consolidating properties, stone-specific studies concerning the compatibility and durability must be done before applying the nanoconsolidant-based treatments to a given monumental stone.

## Supporting Information Available

Details of force measurements between two mica surfaces; DLVO force calculations; supplementary friction data; supplementary X-SFA data; supplementary SEM data; tensile strength calculations; capillary and vdW forces comparison (PDF)
