# Supplementary material for: Cohesion Gain Induced by Nanosilica Consolidants for Monumental Stone Restoration
Source: Langmuir. 2022 May 23;38(22):6949–58. doi: 10.1021/acs.langmuir.2c00486 (PMC9178914; doi:10.1021/acs.langmuir.2c00486)
Supplement: Supplementary file 1 — la2c00486_si_001.pdf [file la2c00486_si_001.pdf]

Supporting Information for:

Cohesion gain induced by nanosilica consolidants for monumental stone restoration

Joanna Dziadkowiec,[1,2,a] Hsiu-Wei Cheng,[2] Michael Ludwig,[3] Matea Ban,[4] Timon Pascal Tausendpfund,[5] Regine von Klitzing,[3] Markus Mezger,[5,6] and Markus Valtiner[2]

[1)] *NJORD Centre, Department of Physics, University of Oslo, Oslo 0371, Norway*

[2)] *Institute of Applied Physics, Applied Interface Physics, Vienna University of Technology, Vienna 1040, Austria*

[3)] *Soft Matter at Interfaces, Department of Physics, Technical University of Darmstadt, 64289 Darmstadt, Germany*

[4)] *Materials Testing Institute, University of Stuttgart, 70569 Stuttgart, Germany*

[5)] *Max Planck Institute for Polymer Research, 55128 Mainz, Germany*

[6)] *Dynamics of Condensed Systems, Department of Physics, University of Vienna, 1090 Wien, Austria*

[a)] Electronic mail: Corresponding author: joanna.dziadkowiec@mn.uio.no

I. SUPPLEMENTARY DATA CORRESPONDING TO MICA-MICA FORCE MEASUREMENTS IN FIGURE 2

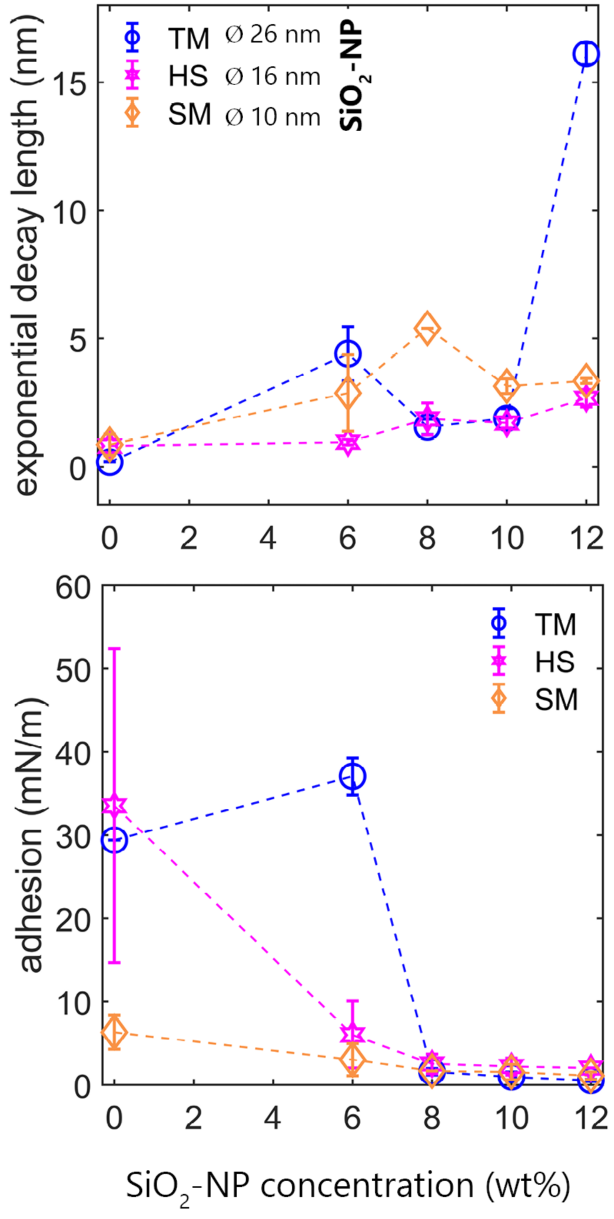

Figure **S1**. Top: Exponential decay lengths and Bottom: adhesion extracted from the SFA force-distance curves measured between two mica surfaces fully immersed in silica nanoparticle suspensions (SiO_2-NP) as a function of silica concentration.The shown measurements correspond to the data presented in Figure 2 in the main manuscript text.

II. CALCULATION OF DLVO FORCES ACTING BETWEEN TWO MICA SURFACES IN WATER

The DLVO forces (F_{DLVO}) for two mica surfaces in MilliQ water plotted in **Figure 2a** in the main manuscript file were calculated as a sum of van der Waals (F_{vdW}) attraction and electrical double layer (F_{EDL}) repulsion:

$$F_{DLVO} = F_{vdW} + F_{EDL} \tag{S1}$$

vdW forces were calculated as a function of surface separation (D) using Hamaker constant (A = $2.2 \cdot 10^{-20}$ J) for mica surfaces across water repored by Pashley et al.[1] in a crossed-cylinder geometry using equations reported by Israelachvili[2]:

$$F_{vdW} = \frac{-A}{6 \cdot D^2} \tag{S2}$$

The EDL repulsion as a function of surface separation (D) was estimated using linear superposition approximation[2] with mica surface potential ψ_0 chosen as -150 mV and Debye length (κ^{-1}) in MilliQ water of 43 nm, assuming ionic strength of $5 \cdot 10^{-5}$ M as in supporting information in Bilotto et al.[3]:

$$F_{EDL} = \kappa \cdot Z \cdot e^{-\kappa D} \tag{S3}$$

$$Z = 64 \cdot \pi \cdot \varepsilon_0 \cdot \varepsilon \cdot (kT)^2 \cdot tanh(\frac{z \cdot e_c^2 \cdot \psi_0}{4kT})^2 \tag{S4}$$

$$\kappa = \sqrt{\sum_i \frac{C_i \cdot e_c^2 \cdot z_i^2}{\varepsilon_0 \cdot \varepsilon \cdot kT}} \tag{S5}$$

where ε_0 is vacuum electrical permittivity [F/m], ε is dielectric constant of pure water, k is Boltzmann constant, T is temperature [K], z is valency of an ion *i*, e_c is elementary electric charge [C], and C_i is bulk molar concentration of an ion *i*.

III. SUPPLEMENTARY FRICTION DATA

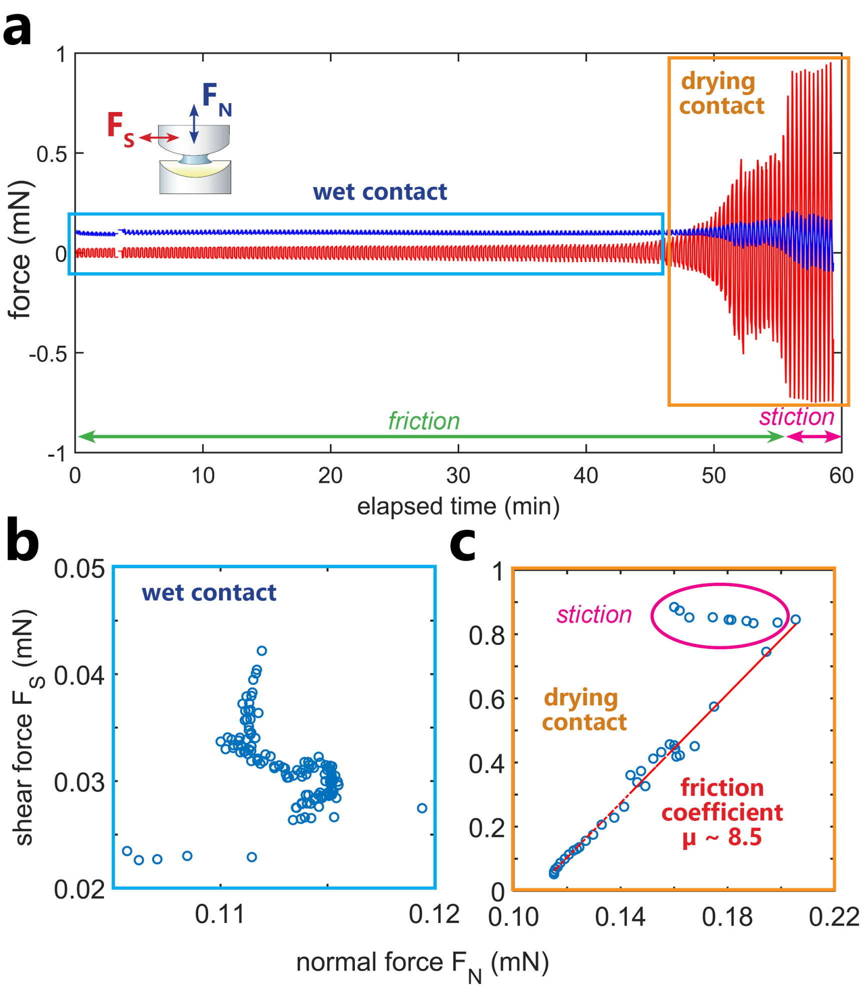

Figure **S2**. Complementary friction data collected during silica 6 wt% Ludox SM droplet drying experiment shown in **Figure 3a**: **a)** Full friction pattern with time elapsed from ∼3 μl silica droplet injection. **Figure 3a** in the main manuscript shows only data after 46 minutes of the drying process. The friction (wet contact) and stiction (with dried silica aggregates) regimes are indicated; **b)** Shear force F_S plotted as a function of normal load force F_N for wet contact as indicated in the subplot **a**. Both F_S and F_N vary little during the initial 46 minutes of silica drying. Due to almost no variation of the F_N, friction coefficient cannot be determined; **c)** Shear force F_S plotted as a function of normal load force F_N for drying and dried contact as indicated in the subplot **a**. The friction coefficient μ ∼8.5 was determined for the final drying stage (excluding the stiction regime) as a slope of the linear fit to the F_S vs. F_N curve, according to the Amontons law. In the final stiction regime, μ is not defined.

IV. SUPPLEMENTARY X-RAY SURFACE FORCES APPARATUS (X-SFA) DATA

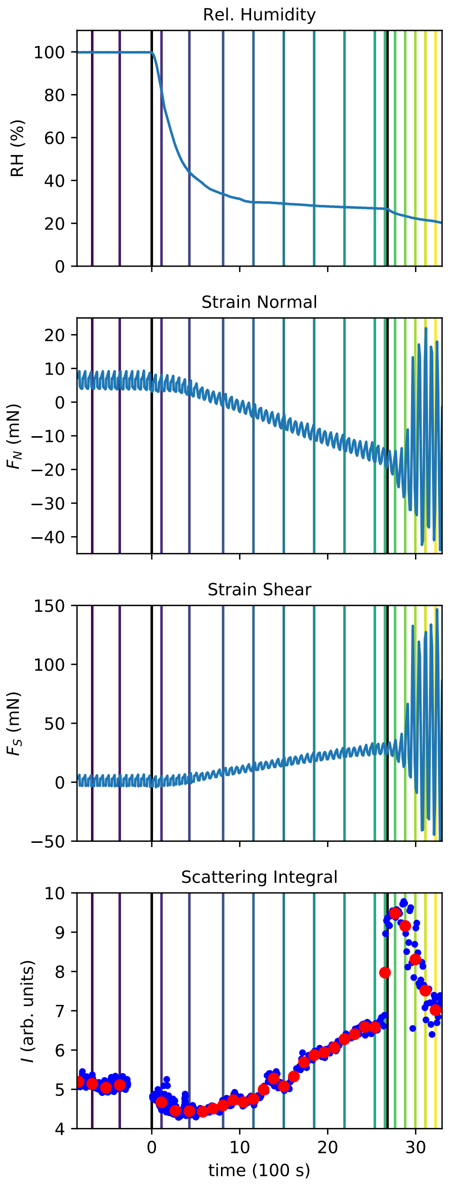

Figure **S3**. Complementary experimental parameters collected during the X-SFA measurements (**Figure 3b,c**) on the 12 wt% Ludox SM droplet drying as a function of elapsed time after purging the sample chamber with dry N_2. Top panel: relative humidity (RH) in the X-SFA chamber; middle panels: normal and shear forces; bottom panel: integrated X-ray scattering signal within an azimuthal angle of ± 30. Radial averages I(q) and 2D data I(q_x, q_y) shown in **Figure 3b,c** were obtained by summation (red circles) of individual 2D data frames (blue circles) corresponding to a total x-ray exposure of 100 s. Colours of vertical lines correspond to timestamps (A-P) of I(q) in **Figure 3b**.

V. SUPPLEMENTARY SCANNING ELECTRON MICROSOPE DATA

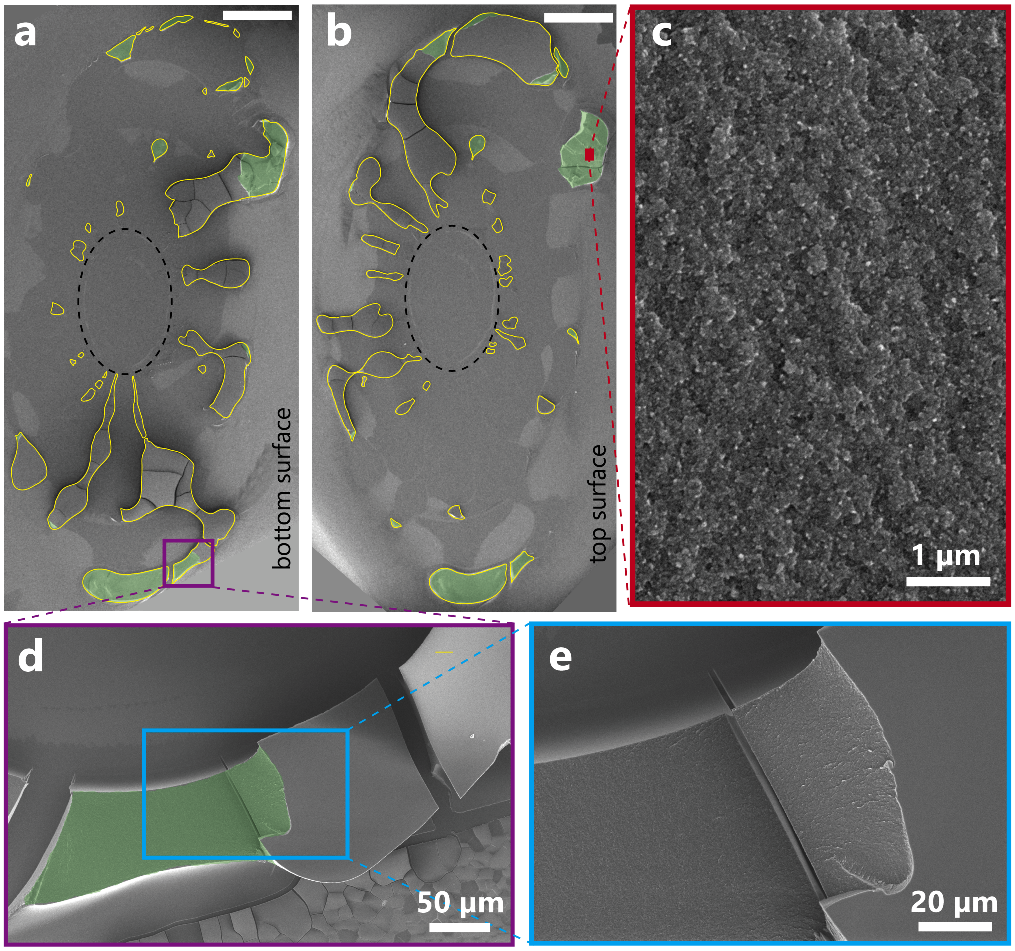

Figure **S4**. Scanning Electron Microscopy images of dried and broken silica bridges on the bottom **a)** and top **b)** mica surfaces used in the SFA breaking experiment. Dashed yellow line outlines all dried silica deposits. Green areas (common for the bottom and top surfaces) shade the silica bridges that broke in the middle, the rest of them detached from one of the mica surfaces. The scale bars are 100 μm. Panels **c)**, **d)**, and **e)** zoom on rough surfaces of the silica bridges that broke in the middle (were not detached from the mica surface on any side).

VI. TENSILE STRENGTH CALCULATIONS

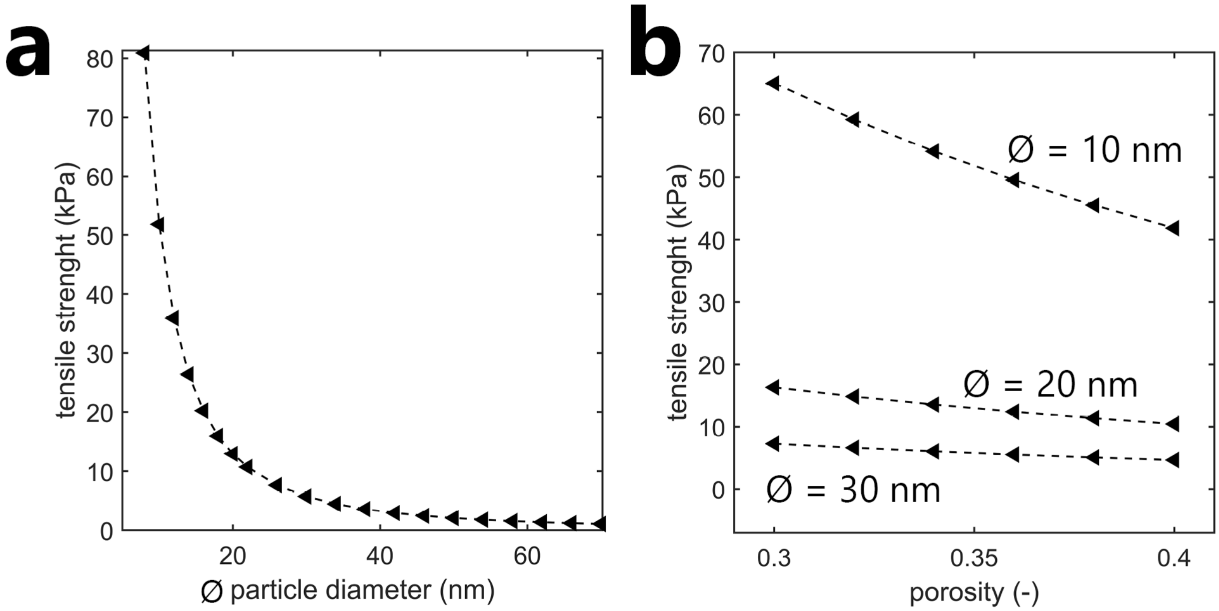

Figure **S5**. Tensile strength (σ) of an aggregate composed of randomly packed, monosized, hard spheres with the particle diameter (ϕ), calculated according to equations provided by Rumpf (1974)[4] and Schubert et al. and[5]; **a)** tensile strength as a function of particle diameter (ϕ) for aggregate porosity of 0.35; **b)** tensile strength as a function of aggregate porosity (Φ) for three different particle diameters of 10, 20, or 30 nm.

Tensile strength (σ) of an aggregate composed of randomly packed, monosized, hard spheres plotted in **Figure S5** was calculated using equations adapted from Rumpf[4] and Schubert[5]:

$$\sigma = \frac{1-\Phi}{\Phi}\frac{F}{R_p^2} \tag{S6}$$

where (Φ) is aggregate porosity and F is attractive interparticle force of a single particle-particle contact. For simplicity, we assume that the total interparticle force F acting between a pair of nanoparticles is maximum capillary force (F_{cap}) at a surface separation D of 0.1 nm, calculated according to the equations derived by Rabinovich et al.[6] (see equations 18 and 20 therein) for two interacting spheres with a particle radius R across a capillary bridge with a fixed volume (V) as a function of surface separation (H):

$$F_{cap}(H,V) = -\frac{2\pi \cdot R \cdot \gamma \cdot cos\theta}{1+[H/2 \cdot d_{sp,sp}(H,V)]} \tag{S7}$$

$$d_{sp,sp}(H,V) = (H/2)\cdot(-1+\sqrt{1+2V/(\pi \cdot R \cdot H^2)}) \tag{S8}$$

where γ is air-water interfacial tension equal to 0.07 N/m, θ is water-silica surface contact angle assumed to be 5 (0.0873 rad), and $d_{sp,sp}$(H,V) is a difference in maximum and minimum height of the liquid capillary bridge across the sphere-sphere contact. Based on experimental measurements of Rabinovich et al.[6], we assume the liquid volume in a capillary bridge between two nanoparticles to be on the order of $5 \cdot 10^{-24}$ m^3.

VII. CAPILLARY AND VAN DER WAALS FORCES COMPARISON

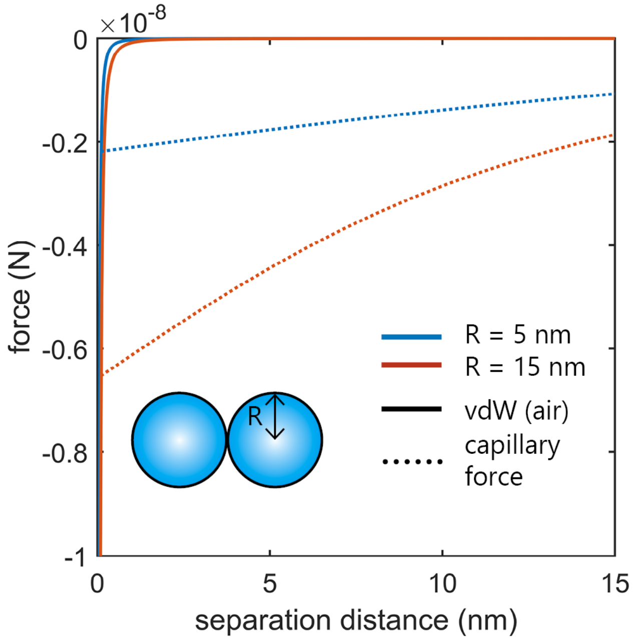

Figure **S6**. Van der Waals and capillary forces calculated for two silica spheres with a radius (R) of 5 or 10 nm.

Capillary forces for two spherical particles plotted as a function of separation distance in **Figure S6** were calculated according to equations nr S7 and S8 provided by Rabinovich et al.[6], as explained in the previous section of the SI. van der Waals attractive forces $F_{vdW,silica}$ acting between two spherical silica particles with a radius R in air were calculated as a function of surface separation D according to equation provided by Israelachvili[2]:

$$F_{vdW,silica} = \frac{-A}{6 \cdot D^2} \cdot \frac{R^2}{2R} \tag{S9}$$

where A is Hamaker constant for two silica surfaces interacting across air of $6.5 \cdot 10^{-20}$ J, provided by Bergström[7]. vdW forces acting between silica particles across water would be an order of

magnitude smaller with Hamaker constant for two silica surfaces across water of $0.46 \cdot 10^{-20}$ J (also provided by Bergström[7]).

REFERENCES

[1] R. Pashley, "Hydration forces between mica surfaces in aqueous electrolyte solutions," *Journal of colloid and interface science*, vol. 80, no. 1, pp. 153–162, 1981.

[2] J. N. Israelachvili, *Intermolecular and surface forces*. Academic press, 2015.

[3] P. Bilotto, M. Lengauer, J. Andersson, U. Ramach, L. L. Mears, and M. Valtiner, "Interaction profiles and stability of rigid and polymer-tethered lipid bilayer models at highly charged and highly adhesive contacts," *Langmuir*, vol. 35, no. 48, pp. 15552–15563, 2019.

[4] H. Rumpf, "Die wissenschaft des agglomerierens," *Chemie Ingenieur Technik*, vol. 46, no. 1, pp. 1–11, 1974.

[5] H. Schubert, "Tensile strength of agglomerates," *Powder Technology*, vol. 11, no. 2, pp. 107–119, 1975.

[6] Y. I. Rabinovich, M. S. Esayanur, and B. M. Moudgil, "Capillary forces between two spheres with a fixed volume liquid bridge: theory and experiment," *Langmuir*, vol. 21, no. 24, pp. 10992–10997, 2005.

[7] L. Bergström, "Hamaker constants of inorganic materials," *Advances in colloid and interface science*, vol. 70, pp. 125–169, 1997.
